# Genome-wide SSR-based association mapping for fiber quality in nation-wide upland cotton inbreed cultivars in China

**DOI:** 10.1186/s12864-016-2662-x

**Published:** 2016-05-13

**Authors:** Xinhui Nie, Cong Huang, Chunyuan You, Wu Li, Wenxia Zhao, Chao Shen, Beibei Zhang, Hantao Wang, Zhenhua Yan, Baoshen Dai, Maojun Wang, Xianlong Zhang, Zhongxu Lin

**Affiliations:** National Key Laboratory of Crop Genetic Improvement, College of Plant Sciences & Technology, Huazhong Agricultural University, Wuhan, 430070 Hubei China; Cotton Research Institute, Shihezi Academy of Agriculture Science, Shihezi, 832011 Xinjiang China; Economic Crop Research Institute, Henan Academy of Agricultural Sciences, Zhengzhou, 450002 Henan China

**Keywords:** Upland cotton, Population structure, Elite allele, Association mapping, Fiber quality traits

## Abstract

**Background:**

Since upland cotton was introduced into China during the 1920s–1950s, hundreds of inbreed cultivars have been developed. To explore the molecular diversity, population structure and elite alleles, 503 inbred cultivars developed in China and some foreign cultivars from the United States and the Soviet Union were collected and analyzed by 494 genome-wide SSRs (Simple Sequence Repeats).

**Methods:**

Four hundred and ninety-four pairs of SSRs with high polymorphism and uniform distribution on 26 chromosomes were used to scan polymorphisms in 503 nation-wide upland cottons. The programming language R was used to make boxplots for the phenotypic traits in different environments. Molecular marker data and 6 fiber quality traits were analyzed by the method of MLM (mixed linear model) (P + G + Q + K) in the TASSEL software package on the basis of the population structure and linkage disequilibrium analysis. The loci of elite allelic variation and typical materials carrying elite alleles were identified based on phenotypic effect values.

**Results:**

A total of 179 markers were polymorphic and generated 426 allele loci; the population based on molecular diversity was classified into seven subpopulations corresponding to pedigree origin, ecological and geographical distribution. The attenuation distance of linkage disequilibrium dropped significantly up to 0–5 cM. Association mapping for fiber quality showed that 216 marker loci were associated with fiber quality traits (*P* < 0.05) explaining 0.58 % ~ 5.12 % of the phenotypic variation, with an average of 2.70 %. Thirteen marker loci were coincident with other studies, and three were detected for the same trait. Seven quantitative trait loci were related to known genes in fiber development. Based on phenotypic effects, 48 typical materials that contained the elite allele loci related to fiber quality traits were identified and are widely used in practical breeding.

**Conclusions:**

The molecular diversity and population structure of 503 nation-wide upland cottons in China were evaluated by 494 genome-wide SSRs, and association mapping for fiber quality revealed known and novel elite alleles. The molecular diversity provides a guide for parental mating in cotton breeding, and the association mapping results will aid in the fine-mapping genes related to fiber quality traits and facilitate further studies on candidate genes.

**Electronic supplementary material:**

The online version of this article (doi:10.1186/s12864-016-2662-x) contains supplementary material, which is available to authorized users.

## Background

Cotton is one of the world’s most important cash crops, and cotton fiber provides an important raw material for the textile industry. There are four cultivated cotton species, including diploids of *Gossypium herbaceum* and *G. arboreum* and tetraploids of *G. hirsutum* and *G. barbadense. G. hirsutum* cottons (upland cottons) are planted widely due to their wide adaptability and high yield. These cottons account for more than 95 % of the world’s cotton production [1].

China is one of the largest cotton producing countries in the world but is not the country of origin for cotton. Cotton production and breeding were developed on the basis of introduced varieties in China [2]. Upland cotton is native to Central America. Trice, Lone star, Stoneville 2B, DPL15, Uganda, KK1543 and 611Bo have been introduced into China from the United States and the former Soviet Union since 1918 [3]. Cotton breeding in China experienced seven breeding generations, new varieties updates (1904–1920–1936–1948, 1949–1958, 1959–1964, 1964–1979, 1980–1984, 1980s–1990s, the middle and later periods of 1990s-now), and expanded planting area. Recently, thousands of upland cotton varieties and lines have been domesticated, bred and derived, in which more than 200 good varieties are widely employed in production. Thus, the five main cotton regions, including the Yellow River Region (YRR), the Yangtze River Region (YtRR), the Northwestern Inland Region (NIR), the Northern Specific Early Maturation Region (NSEMR) and the Southern China Region (SCR) formed gradually [4]. Due to the narrow genetic basis and long-term directional selection in breeding, the genetic diversity in these upland varieties is low [5–9]. Therefore, the study on genetic diversity of basic upland germplasms and derived varieties can reveal the cotton genetic basis in China, provide understanding of the genetic background and genetic diversity of existing germplasms, lay the foundation for effectively exploring and using genes of important traits for breeders, and ascertain the direction of germplasm innovation.

The majority of traits in crops, such as agronomy, yield, quality and resistance, belong to quantitative traits controlled by multiple genes and present continuous phenotypic variation in segregation populations. The quantitative trait loci (QTL) with minor contributions to trait phenotype and sensitivity to environments lead to difficulties of identifying them [10]. Recently, the development of molecular markers and the rapid development of statistical analysis methods for quantitative traits have provided a platform for the genetics of crop quantitative traits. With the increase of molecular marker and the release of cotton genome sequences, cotton genetic maps have become increasingly saturated [11, 12], and QTL have been identified for agronomic traits [13], fiber quality [14, 15], growth stages [16] and resistant traits [17–21] by linkage mapping. However, linkage mapping has its own limitations: the segregation populations are from two specific parents, and linkage mapping only refers to two alleles at the same loci; the limited number of reorganization events occurring in gene loci leads to QTL with low resolution, the precision of the linkage analysis is commonly up to 10–30 cM; the QTL detected in specific genetic backgrounds and environments cannot be extensively applied in other hybrid combinations and the environment, which should be further verified.

In recent years, exploring quantitative trait genes by association analysis has been one of the most active research topics in plant quantitative genetics. Association analysis, also known as linkage disequilibrium mapping or association mapping, is based on linkage disequilibrium and combines analyzing the diversity of target traits and gene (locus) polymorphism to identify marker loci with the functions of specific genes closely related to phenotypic variation. Association analysis offers the following advantages compared with traditional linkage analysis: taking the natural population as the experimental materials, detecting multiple alleles on the same locus and targeting single genes. However, obvious complements exist between linkage and association analysis with respect to the accuracy and breadth of QTL mapping, the amount of information and statistical analysis methods. Linkage analysis preliminarily locates the allele controlling a target trait; association analysis performs fast fine-mapping of the target gene [22]. Thus, it is necessary to combine these advantages to confirm the QTL by linkage analysis.

In cotton, researchers has been conducted on traits related to agronomy, fiber quality, yield, growing stage and resistance using association analysis, and multiple marker loci associated with the above traits, elite alleles and carriers for breeding materials [23–25] have been identified. However, the materials were limited in these studies, which originated from limited cotton regions whose representations were not sufficient. The markers used in association analysis did not uniformly distribute on each chromosome, so they could not cover the whole cotton genome.

In this study, 503 upland cotton inbred cultivars, including those that have been grown in China since 1918 and inbred cultivars developed between 1920 and 2011, were used as the population panel; 494 genome-wide SSR markers from our high-density interspecific genetic map with 5152 markers [12] were selected at an average 10 cM to genotype the population. The objectives of our study were: (1) to analyze the population structure of upland cotton inbred cultivars developed in China; (2) to detect the marker loci associated with fiber quality traits; (3) to explore the elite alleles and the typical carried materials for future molecular design breeding in cotton; and (4) to provide multiple candidate genes and lay a foundation for further fine-mapping and gene cloning.

## Results

### Molecular genetic diversity

Among the 494 genome-wide SSR markers, 179 primer pairs displayed polymorphism, accounting for 36.16 % of the total primers, with an average of 6.885 markers per chromosome. A total of 426 allele loci were detected, with an average of 2.379 alleles per marker (ranging from 1 to 8). The average number of genotypes per marker was 4.413 (ranging from 2 to 34). The average genetic diversity was 0.377 (ranging from 0.012 to 0.893). The average polymorphism information content (PIC) was 0.336 (ranging from 0.012 to 0.887) (Additional file 1).

The average genetic similarity coefficient variation among the 503 cultivars was 0.552 (ranging from 0.337 to 0.921) (Additional file 2). A two-dimensional diagram of the principal coordinate (PCA) analysis was produced based on the genetic distance (GD) matrix. From axis 1 to 3, the percentage of explained variance of individual was 31.36 %, 22.24 %, and 13.27 %, respectively. The variation among subpopulations accounted for only 4 % of the total variance and variation within subpopulations accounted for 96 % (Table 1).

**Table 1:** AMOVA of the populations (pops)

Source	*df*	SS	Est. Var.	Percentage of variance	*P*-value
Among Pops	6	1160.151	2.396	4	<0.001
Within Pops	496	27596.634	55.638	96	<0.001
Total	502	28756.785	58.007	100	

### Population structure

**Fig. 1:**
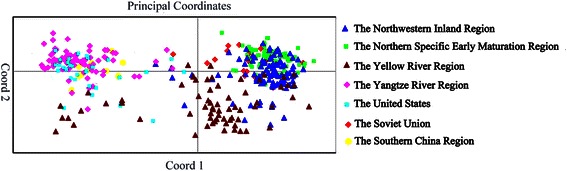
PCA plots of 503 upland cotton cultivars based on SSRs. Blue triangles, green squares, brown triangles, purple diamonds, light blue square, red diamonds and yellow circles represent cultivars from different regions

Three methods were used to determine the population structure. First, the genetic structure based on SSR markers was constructed by separating PCA plots, which revealed that the population was divided into 7 groups (Fig. 1). The results revealed that each group was relatively independent, but there was mutual fusion. The special characteristics in each cotton region were formed due to the unique climate and geographical ecological environment. For example, the early-medium maturity cotton varieties cultivated in dense planting were more suitable for the NIR and the NSEMR. Additionally, varieties in each cotton region, which were exchanged with each other, formed the same pedigree source. For example, as summarized in Additional file 3: Table S2, ZY10 served as a parent for ZY478 (NIR), ZY459 and ZY303 (YRR), ZY398 (YtRR). Thus, the cultivars in different cotton regions exchanged and maintained a relatively open system.

Secondly, based on Nei’s genetic distance, the population formed 7 distinct groups in the unrooted tree (Additional file 4: Figure S2a), including 106, 19, 147, 67, 9, 103, and 52 cultivars in Groups I to VII, respectively. Combining the genealogical, geographical and ecological distribution, each group was composed of cultivars from different sources but was dominated by cultivars from the same cotton area (Additional file 4: Figure S2b).

**Fig. 2:**
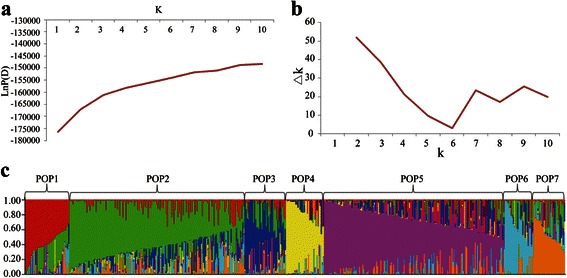
Population structure analysis and lines chart of the K value with LnP(D) value and ΔK value based on structure analysis. Line chart of the LnP(D) value with the change of K (**a**); Line chart of the ΔK value with the change of K (**b**); Population structure of 503 cultivar-based SSR markers (**c**). *Q*-plot showing the clustering of 503 upland cotton cultivars based on the analysis of genotypic data using STRUCTURE. Each cultivar is represented by a *vertical bar*. The colored subsections within each *vertical bar* indicate the membership coefficient (*Q*) of the cultivar to different clusters

Thirdly, the population structure was analyzed using STRUCTURE software. The K value increased continuously with the increase of the LnP(D) value, and no such plateau or obvious upward inflexion point was reached in this panel (Fig. 2a). As shown in Fig. 2b, although the ΔK value decreased rapidly from K = 2 to K = 5, K = 7 represented the first peak (upward inflexion point), indicating that the population structure could be divided into 7 subgroups. The 7 subgroups included 79, 141, 28, 20, 225, 6 and 4 cultivars (Fig. 2c). Thus, based on the three clustering methods, this population should be classified into 7 subpopulations.

### Linkage disequilibrium

**Fig. 3:**
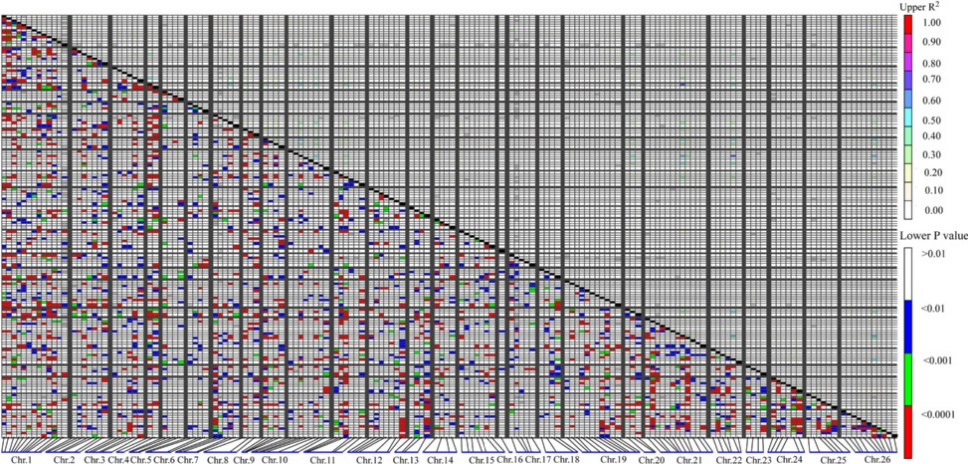
The distribution of LD among 426 SSR loci on 26 chromosomes in 503 upland cotton cultivars (R^2^ value). SSR markers were along the X-axis. Each pixel above the diagonal represents the r^2^ size of the corresponding pairs of markers, as shown in the color code at the upper right, and each pixel below the diagonal represents the P-value size of testing the LD at the lower right

The linkage disequilibrium (LD) of this population was analyzed using 179 SSR markers. In a total of 11628 pairwise comparisons of 426 polymorphic SSR marker loci, 27.71, 17.26 and 14.51 % of SSR marker loci demonstrated significant LD at *P* < 0.05, *P* < 0.01 and *P* < 0.005, respectively. Based on r^2^ estimates, only 2.09 % (r^2^ ≥ 0.05) and 1.30 % (r^2^ ≥ 0.1) of the marker pairs showed significant LD. In addition, the LD distribution was unevenly distributed on each chromosome, where the loci of higher LD level dramatically concentrated on chromosome01, 02, 15, 19, 21, 24 and 26 (Fig. 3).

To identify the genome-wide LD decay, r^2^ and D’ values of LD were plotted as a function of genetic distance in cM. The significant pairwise LD (r^2^  ≥0.05) was observed between some SSRs loci pairs within 50 cM distance. The genetic distance within 0–25 cM rapidly reduced when genome-wide LD was r^2^  ≥0.018 (Additional file 5: Figure S3a). Thus, genome-wide LD at r^2^ < 0.03 (Additional file 5: Figure S3b) and D’ = 0.25 (Additional file 5: Figure S3c) was reduced to 0–5 cM, revealing potential for association mapping.

### Phenotypic variation of fiber quality traits

The phenotypic data (Additional file 6) of fiber quality in eight environments were determined by best linear unbiased prediction (BLUP), and then the breeding value of each cultivar for six fiber quality traits was obtained for association analysis. The cotton cultivars from seven cotton ecological regions in this study represented a broad variation in each experiment site. The highest coefficient of variation in FUHML (5.410 %) and FU (1.098 %) was discovered in cultivars from the Soviet Union; FS (6.168 %) and MV (6.394 %) from the NIR; SF (10.201 %) and FE (10.669 %) from the NSEMR and the United States, respectively. The highest coefficient of variation was observed in FE (9.31 %), the lowest in FU (0.80 %). The heritability was higher in FUHML and FE (0.93 and 0.91), ranging 0.84 to 0.88 in the other five traits (Additional file 7).

The correlations of six fiber quality traits using the results of BLUP processing were listed in Additional file 8, and highly significant correlations were observed among the six fiber quality traits. There were positive correlations between FUHML and FS and FU and between MV and FE. There were negative correlations between FUHML and MV, FE, and SF; FS and FE and SF; and FU and SF and FE.

**Fig. 4:**
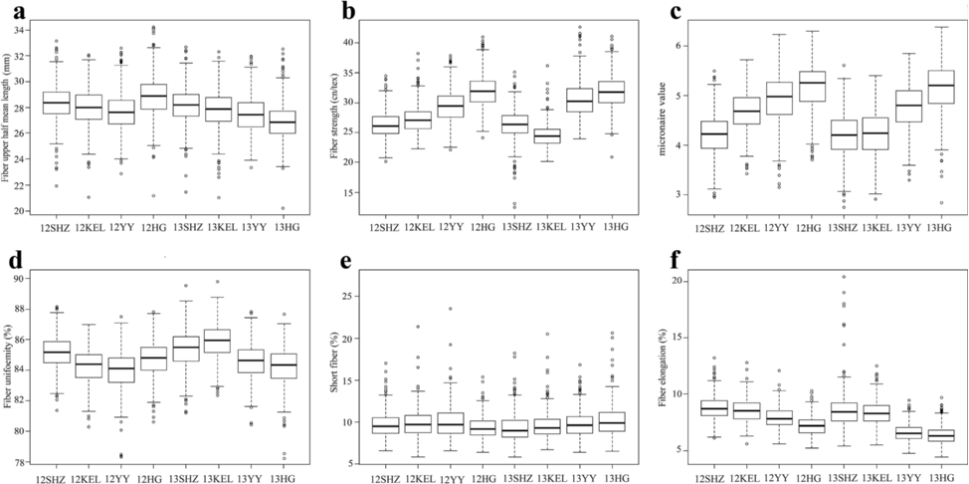
The boxplots of the changing trends of six fiber quality traits in eight environments

The phenotype trends of fiber quality are shown in Fig. 4. FUHML (Fig. 4a), FS (Fig. 4b), MV (Fig. 4c) and FE (Fig. 4f) had relatively stable changing trends in eight environments. The trait changing trends of FU (Fig. 4d) and SF (Fig. 4e) were less stable in the eight environments. For instance, in 2012 and 2013, the means of FU in Kuerle were 84.51 % and 85.91 %, respectively, with increasing trends, whereas they were 84.85 % and 84.01 %, in Huanggang, with decreasing trends (Fig. 4d).

**Fig. 5:**
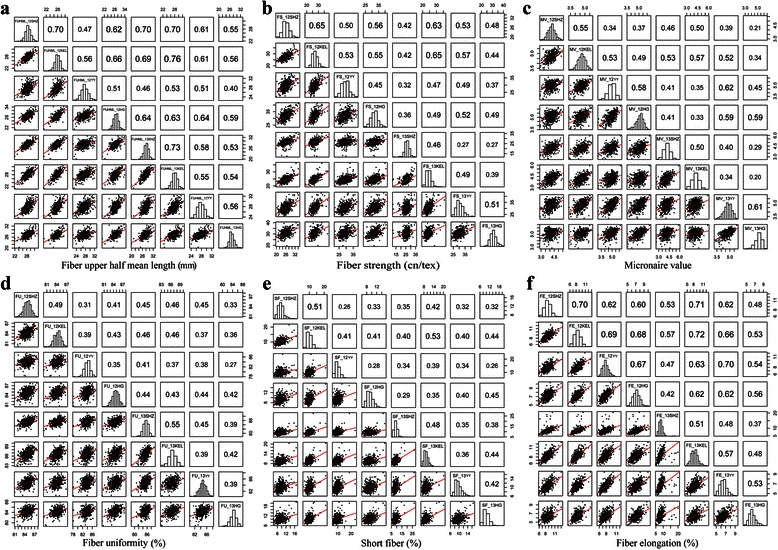
The correlations of six fiber quality traits in eight environments

The correlations between two environments were obtained among eight environments for the six fiber quality traits (Fig. 5). Among the six fiber quality traits, the correlation means were ordered as FUHML (0.593) > FE (0.581) > FS (0.474) > MV (0.445) > FU (0.410) > SF (0.380). It was more important to further analyze one trait between two environments; taking FUHML as example (Fig. 5a), the correlations ranged from 0.40 to 0.76, and the correlation was 0.76 for FUHML_12KEL and FUHML_13 KEL. The red line that was near the 45° as the line of greatest slope indicated a correlation between FUHML_12KEL and FUHML_13KEL.

### Association mapping of fiber quality-related traits

Based on the genotype data, the PCA matrix, the kinship matrix, and the fiber quality traits data of the BLUP results in 8 environments, a mixed linear model was used to analyze the marker-trait associations. During association mapping, three models, GLM (P + G) + Q, GLM (P + G) + PCs, and MLM (G + P + Q + K), were compared with each other in the association analysis (Additional file 9). The control effect of the population structure for FUHML, FS, SF, and FE in the three models was similar. However, the MV analysis using the MLM-Q-K model was superior to the other two models, and the FU analysis using the GLM-Q and MLM-Q-K models was better than the of GLM-PCA model. According to the above comparison results, the MLM-Q-K model had better performance.

**Fig. 6:**
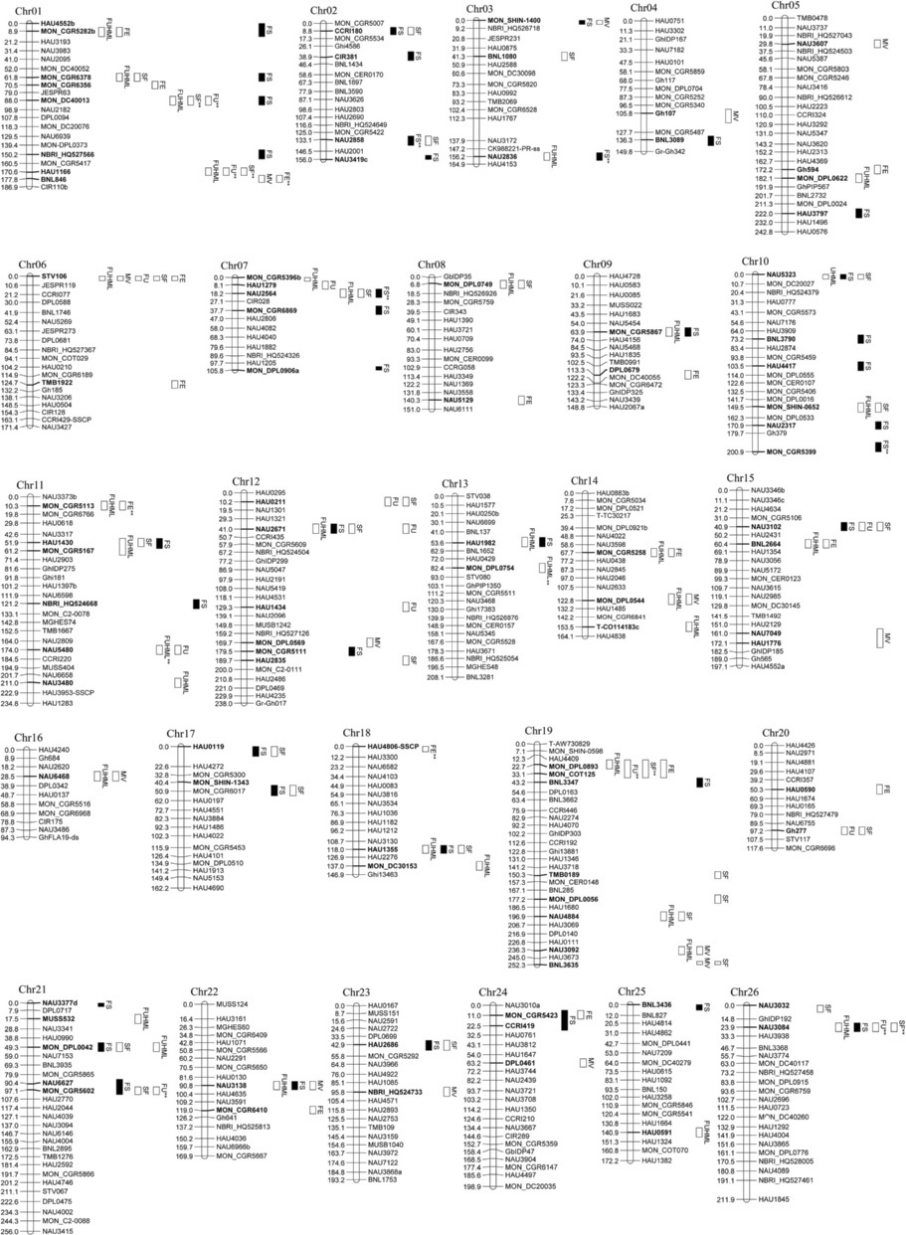
The distribution of the located markers associated with the fiber quality-related traits on 26 chromosomes

A total of 179 SSR markers were used for marker-trait association after filtering for 5 % minimum alleles, among which 91 (50.84 %) markers were associated with fiber quality traits at the *P* < 0.05 level. Fifteen markers were significantly associated at the *P* < 0.01 level (Fig. 6). An average of 3.5 markers was detected on each chromosome (ranging from 1 to 8), with the maximum of 8 markers on Chr01 and Chr19. One marker was generally associated with several traits. For example, MON_DC40013 on Chr01 was related to FUHML, SF, FU and FS, and NAU2564 on Chr07 was related to FUHML, SF and FS.

There were 216 loci associated with fiber quality components at the *P* < 0.05 significance level, among which 27 were significant at the *P* < 0.01 level. The range of phenotypic variation explanation (PVE) observed was from 0.58 % (MON_DPL0042b) to 5.12 (NAU3084c), with an average of 2.70 % (Additional file 10).

Among the 6 traits, FS was associated with the most loci, up to a maximum of 61 (*P* < 0.05) and 7 (*P* < 0.01); PVE ranged from 0.58 (MON-DPL0042b, *P* < 0.05) to 3.17 % (NAU2836a, *P* < 0.001), with a mean of 2.63 %. The remarkable contribution loci were NAU2858a (2.92 %), BNL3089a (2.86 %) and MONCGR5399c (2.73 %), especially at the *P* < 0.001 level.

FUHML was associated with the second number of loci, up to a maximum of 46 (*P* < 0.05) and 4 (*P* < 0.01); PVE ranged from 0.59 (NAU3092a, *P* < 0.05) to 3.11 % (NAU5480b, *P* < 0.001), with a mean of 2.35 %, in which NAU5480a (2.75 %) had a significant contribution at *P* < 0.001.

There were up to 42 (*P* < 0.05) and 6 (*P* < 0.01) loci associated with SF, and PVEs ranged from 0.68 (HAU2835b, *P* < 0.05) to 5.12 % (NAU3084c, *P* < 0.001), with a mean of 3.80 %. NAU3084b (5.08 %), MON-DPL0893a (4.43 %), and MON-DC40013b (3.03 %) contributed prominently to SF at *P* < 0.001.

There were 25 (*P* < 0.05) and 3 (*P* < 0.01) loci associated with FE, with PVE ranging from 0.70 (MON-CGR5423b, *P* < 0.05) to 2.99 % (HAU4806-SSCPa, *P* < 0.001). BNL846b and MON_CGR5113b contributed to FE at *P* < 0.01.

There were 23 (only at *P* < 0.05) loci associated with MV, with PVE ranging from 0.66 (NAU3138b) to 1.46 % (DPL0457a); NBRI-HQ524733b contributing to MV was detected at *P* < 0.05.

There were 19 (*P* < 0.05) and 7 (*P* < 0.01) loci associated with FU, and the PVEs ranged from 1.06 (HAU1279d) to 2.51 % (HAU1166a), with a mean of 2.14 %. The contribution loci were observed in MON-CGR5602a (2.39 %), MON-DPL 0893a (2.26 %), MON-DC40013b (2.21 %), NAU3084c (1.93 %), HAU1166b (1.87 %) and NAU3084b (1.82 %) at *P* < 0.01.

### Exploring elite allele-related genes in the cotton genome

The reference sequences of 91 elite allele loci associated with fiber quality traits were explored based on related genes in *G. arboreum*, *G. raimondii* and *G. hirsutum*,.

Three allelic variation loci were related to gene functional annotation of fiber quality traits in *G. arboreum* (Additional file 11). HAU0211 was associated with FU and SF on Chr12; its homologous genes in *G. arboreum* and *Arabidopsis thaliana* were Cotton_A_01461 and AT5G16560.1, respectively, which were annotated as Home-domain-like HD-ZIP family with the function of promoting cotton fiber elongation and initiation. HAU1355 was associated with FUHML, FS and SF on Chr18; its homologous genes in *G. arboreum* and *Arabidopsis thaliana* were Cotton_A_16285 and AT4G32551.2, respectively, which were annotated as WD40 repeat-like-containing domain family with the function of promoting fiber epidermal cell initiation. MON-CGR5167 was associated with FUHML and FS on Chr11; its homologous genes in *G. arboreum* and *Arabidopsis thaliana* were Cotton_A_07705 and AT4G00050.1, respectively, which were annotated as basic helix-loop-helix (bHLH) DNA-binding superfamily protein family to promote fiber epidermal cell initiation.

Five allelic variation loci were related to the gene functional annotation of fiber quality traits in *G. raimondii* (Additional file 12). The homologous genes of BNL3436, associated with FS on Ch25 in *G. raimondii* and *Arabidopsis thaliana*, were Cotton_A_07705 and AT4G00050.1, respectively, with the gene annotation of UDP-glycosy-transferase 73B4, which is involved in cell wall synthesis and fiber development. HAU0211 and HAU1355 were discovered with the same gene functional annotation as in *G. arboreum*. NAU2564 was associated with FUHML, SF and FS on Chr07 with the same gene as MON_CGR5167 in *G. arboreum*. The homologous genes of NAU6627, associated with FS on Chr21, in *G. raimondii* and *Arabidopsis thaliana* were Gorai.007G150900 and AT5G43900.1, respectively, which have gene function related to Myosin 2 and may be connected with the cell skeleton.

Four allelic variation loci were related to the gene functional annotation of fiber quality traits in *G. hirsutum* (Additional file 13). HAU1355, MON_CGR5167 and HAU0211 had the same gene annotation, as described above. STV106, with the homologous genes Gh_A06G0097 and AT1G65910.1 in *G. hirsutum* and *Arabidopsis thaliana*, respectively, was a newly discovered allelic variation loci associated with FUHML, MV, FU, SF and FE on Chr06, whose gene annotation was an NAC domain containing protein 28, which thickens the secondary wall in *Arabidopsis thaliana* and the xylem and cell wall in cotton.

### Discovery of superior alleles and typical materials

According to the genotype data of the loci associated with fiber quality-related traits identified at *P* < 0.05 and the phenotype data of the BLUP results of 6 fiber quality-related traits in 8 environments, 48 materials with superior alleles were discovered (Additional file 14). Taking FUHML as an example, 15 marker loci of positive phenotypic effects and 10 marker loci of negative phenotypic effects were found, with BLUP values ranging from 30.0 to 31.48 mm and from 22.87 to 25.98 mm, respectively. NAU1982a was the allelic variation locus with the maximum positive phenotypic effect (+0.473 mm) in ZY495; meanwhile, MON-CGR6378c was the allelic variation locus with the maximum negative phenotypic effect (−1.23 mm) in ZY83.

## Discussion

### Population construction

The population panel consisted of 503 cultivars including some basic germplasms introduced from abroad and evolved through three variety replacement stages (King, Trice and Lxme star were introduced to the Northern Cotton Regions in 1920s and partially replaced *G. arboreum* varieties; Stoneville4, Delfos531 and DPL14 replaced half of the *G. arboreum* varieties in the 1930s and 1940s; DPL15, Stoneville2B and Stoneville5A replaced *G. arboreum* varieties, which were planted for a long time, and outdated *G. hirsutum* varieties in the 1950s) [26], and breeding varieties from 1911 to 2011 in China. Compared with the sample size of the population in previous researches [24, 27], our population was more comprehensive than others and was larger than 500, which was sufficient for statistical power [22]. The population panel included cultivars from five main representative cultivated cotton regions and was thus enriched with abundant variations in yield, fiber quality and disease resistance. In this study, the evaluation of six fiber quality-related traits in eight environments showed wide variations (0.80 ~ 9.31 %), stable heritability (0.84 ~ 0.93) (Additional file 7), and stable changing trends of each trait in different environments (Fig. 4). Phenotypic traits analysis based on the BLUP results ruled out environmental effects and improved the accuracy of the complex quantitative traits. Both the composition of the population and the trait evaluation indicated that this population panel could be considered as an ideal resource for association mapping of quantitative traits in *G. hirsutum*.

### Molecular genetic diversity

In this study, a total of 179 SSR markers produced 426 allele loci after polymorphism detection from 494 genome-wide SSRs in the population. The average genetic diversity and PIC were 0.377 (ranging from 0.012 to 0.893) and 0.336 (ranging from 0.012 to 0.887) respectively, which were higher than that of Qin et al. [24]. Our research results indicated that the selected markers had sufficient polymorphic information to reveal the genetic relationship between these upland cotton inbred cultivars.

The results also indicated that the average genetic similarity coefficient variation was 0.552 (ranging from 0.337 to 0.921), which could benefit cotton breeders in selecting parents in hybridization breeding to create novel variations and to develop new cultivars. Comparison with previously published studies [5–9, 28] showed that the molecular diversity in our population was higher. In fact, the previous researchers emphasized the narrow genetic basis of upland cotton and selected only upland cotton germplasms representing part of the cotton planted areas or breeding periods. For example, the studies on upland cotton germplasms by Ai et al. [9] and Nie et al. [28] only studied the Northwest Inland cotton varieties (in the north and south of Xinjiang), which were mainly derived from the former Soviet Union.

### Population structure

The evaluation of the population genetic structure is a prerequisite of genome-wide association studies [22], because false associations usually caused by population structure [29]. A reasonably accurate population structure can lead to more genetic similarity within each group and higher genetic differences among groups and largely reduce the defectives in association analysis [30]. Thus, the accuracy of the association analysis depended on whether the population structure was appropriate [31, 32]. Previously, Abdurakhmonov et al. used Q-matrix to estimate accession clusters [23]. Qin et al. [24] and Cai et al. [33] determined the population structure using STRUCTURE software to show K values. Wang et al. [34] determined the genetic structure of 55 accessions of *G. barbadense* by separating PCA plots based on SSR and SRAP markers. In this study, the three methods of PCA, unrooted and rounded tree, with K = 7 corresponding to the uppermost structural level in the contact zone model, were combined to predict the population structure based on 426 allele loci generated by 179 SSR markers distributing on the 26 chromosomes. The population was classified into 7 subpopulations, which was reasonable to eliminate the spurious association effects in the association analysis.

### Linkage disequilibrium

LD, defined as nonrandom combinational alleles at different gene loci [29], is the genetic underpinnings of association analysis. In our study, it was shown that different numbers of linear arrays of linkage disequilibrium loci distributed on each chromosome, especially the pair-loci number of LD on Chr01, 10, 12, 15, 19, 21, and 26 (Fig. 3), on which there were more polymorphic loci associated with fiber quality traits. For example, among the 13 polymorphic loci on Chr01, 8 marker loci were related to fiber quality traits. Therefore, linkage disequilibrium was the basis of the association analysis, in which each pair-loci of LD represented a group allelic variation. The stronger LD degree between the loci in the different allelic variations with functional differences, the more QTL associated with their phenotype were detected.

Recombination was the greatest factor influencing LD. Generally, LD level is high in self-pollinated crops, and low in cross-pollination crops because it will have a higher recombination rate and break the linkage disequilibrium between gene loci [35, 36]. Cotton is a cross-pollination crop with a higher recombination rate; moreover, a significant amount of human behavior, including cross, backcross, open pollination, germplasm introduction and exchange during breeding in different cotton planting regions greatly increase the recombination rate, which leads to the low LD level in the cotton genome. Abdurakhmonov et al. [25] found that the attenuation distance of LD was 25 cM, 10 cM and 30 cM at r^2^ ≥ 0.1, and the distance reached 5–6 cM, 1–2 cM and 6–8 cM at r^2^ ≥ 0.2; Zhang et al. [37] reported that the attenuation distance of LD was 3.4 cM and less than 1 cM at r^2^ ≥ 0.1 and r^2^ ≥ 0.2 respectively. Fang et al. [38] observed that the attenuation distance of LD of the average chromosome was approximately 6.75 cM. Qin et al. [24] discovered that the attenuation distance of linkage disequilibrium decreased dramatically to 0–10 cM. In this study, the attenuation distance of LD decreased dramatically to 0–5 cM, as seen from the LD attenuation figure (Additional file 5), which would be useful to fine mapping candidate QTL.

### Association mapping QTL for fiber quality traits

The 216 marker loci (*P* < 0.05) associated with six fiber traits in this study were compared to other reported QTL in cotton. Thirteen marker loci identified in our study coincided with previous research, of which 3 marker loci were detected with the same traits (Table 2). NAU3419 [39] on Chr02 and related to FS was located at different positions. NAU5480 [40], dramatically significant for FUHML and with much higher explanation of variation, was detected on Chr11. BNL3436 [33] on Chr25, significant for FS, was detected by two previous researchers. The common QTL detected by different researchers indicated that the marker loci associated with fiber quality traits in our study were reliable.

**Table 2:** Comparison of QTL associated with fiber quality traits to the reported studies

Locus	Our research				Other research
	Position(cM)	P-FDR	r^2^	Trait	Trait
CIR381	38.868	4.81E-02	0.89	FS	LI [61]; FE, FL, FS, MIC, FE, MAT, RD, FB [33];
					FL, MIC, MAT, FR, FB [62]; PER, WF, SL [63]
NAU3419	156.034	4.20E-02	1.00	FS	FL, FS, FM [38]; PB [64]
NAU2836	156.228	3.40E-02	1.08	FUHML	FM [65]
		8.69E-04	3.17	FS	
NAU5129	140.322	3.92E-02	1.06	FE	BS [66]
BNL3790	73.2	1.59E-02	1.53	FS	SL [67]
NAU5480	173.978	7.33E-04	3.11	FUHML	FS [68]; FL, FS [40]
		4.85E-02	1.12	FU	
NAU2671	40.995	3.83E-02	1.01	FUHML	SY, LY, BPP [69]
		2.42E-02	1.32	FS	
		3.29E-02	1.48	SF	
		3.87E-02	1.26	FU	
NAU6468	28.487	4.93E-02	0.88	FUHML	FBN [61]
		0.03980	1.11	MV	
HAU0119	0	2.41E-02	1.30	FS	FM [65]
		3.46E-02	1.39	SF	
BNL3347	43.208	2.34E-02	1.34	FS	HP [67]; MIC, FU, SCI [70]
NAU3092	236.322	4.71E-02	0.59	FUHML	LI [64]
		0.03769	0.77	MV	
Gh277	97.156	3.87E-02	1.4	SF	
BNL3436	0	3.45E-02	0.78	FS	FL, FM [71]
NAU3084	23.901	2.68E-02	1.39	FUHML	FB [66]; BW, NB [64]
		1.59E-02	1.51	FS	
		7.34E-03	1.93	FU	
		5.12E-06	5.08	SF	

Comparing our QTL reference sequences to *G. arboreum* [41], *G. raimondii* [42] and *G. hirsutum* [43] showed that 7 QTL were related to the gene function of fiber development (Table 3). Recently, Shan et al. verified that a homeodomain-like superfamily protein, GhHOX3, could control cotton fiber elongation [44], and was associated with the marker locus for fiber length and uniformity [45]. Coincidentally, some genes annotated as the homeodomain-like superfamily in the marker locus HAU0211 were found for fiber uniformity in our study. A gene annotated as UDP-glycosyltranserase was found to be related to BNL3436. UDP-glycosyltransferase was reported to be involved in the regulation of cell wall pectin biosynthesis [46]. These candidate genes within the QTL could affect mature fiber quality traits by regulating fiber cell development and cell wall biogenesis. NAU6627 (r^2^ = 1.89, *P* = 0.0110) and MON-CGR5602 (r^2^ = 1.89, *P* = 0.0366), which were 6.7 cM in genetic distance, were significantly associated with FS on Chr21. NAU6627, related to a gene annotated as myosin2, a cytoskeleton related gene, was within the marker locus BNL3436 for fiber strength. There was a report that another cell skeleton interaction protein, GhWLIM1a, could promoter fiber cell elongation and regulates fiber secondary cell wall biogenesis [47]. Overexpression of the GhWIM1a gene also could increase mature fiber strength, which indicated that myosin2 was an important candidate gene identified from QTL associated with fiber strength. STV106 was related to an NAC domain containing protein [48], which could regulate secondary wall biogenesis and may partially explain why STV106 was associated with five main fiber quality traits. Cotton fiber cell initiation was similar to trichome in Arabidopsis, which is regulated by WD40, bHLH and MYB transcript factors [49]. We found that some genes that were annotated as WD40 or bHLH in HAU1355, MONCGR5167 and NAV2564. The relationships between fiber development-related genes and mature fiber quality traits require further research.

**Table 3:** Allelic variation loci associated with fiber quality and annotated genes

Allelic variation loci	Chromosome	Trait	Position (cM)	P-FDR	r^2^	Gene annotation
HAU0211	12	FU	10.192	3.77E-02	1.24	Homeodomain-like superfamily protein
		SF		4.48E-02	1.14	
HAU1355	18	FUHML	118.042	3.26E-02	0.76	WD40/YVTN repeat-like-containing domain
		FS		1.72E-02	1.08	
		SF		3.40E-02	0.96	
MON_CGR5167	11	FUHML	61.206	3.88E-02	1.04	basic helix-loop-helix (bHLH), DNA-binding superfamily protein
BNL3436	25	FS	0	3.45E-02	0.78	UDP-glycosyltransferase 73B4
NAU2564	7	FUHML	18.223	3.05E-02	1.17	basic helix-loop-helix (bHLH), DNA-binding superfamily protein
		SF		2.71E-02	1.63	
		FS		3.53E-03	2.33	
NAU6627	21	FS	90.434	1.10E-02	1.89	myosin 2
STV106	6	FUHML	0	1.88E-02	1.59	NAC domain containing protein 28
		MV		0.03775	1.19	
		FU		4.59E-02	1.17	
		SF		2.77E-02	1.75	
		FE		4.38E-02	1.06	

### Applications in breeding

The information of this study provided the characteristics of phenotypic variation of fiber quality traits, the genetic diversity, population structure and elite alleles and encouraged us to take a further study to propose a detailed scheme for applying the above results in cotton breeding.

Firstly, specific and elite alleles considered as selection tags of genetic fragments of introgression lines were used to characterize different foreground selection parents and were then crossed within infiltration lines to assemble elite alleles from different marker loci into one recipient parent and breed excellent hybrid progeny by allelic bands assisted selection. For example, in our study, NAU5480 (r^2^ = 3.11), MON_DPL0544b (r^2^ = 1.84) and HAU1166 (r^2^ = 1.82) associated with FUHML,NAU2836 (r^2^ = 3.17) and NAU2317 (r^2^ = 1.64) associated with FS, and DPL0457 (r^2^ = 1.46) and NAU6468 (r^2^ = 1.11) associated with MV. These elite alleles could be combined by marker-assisted selection to develop accessions with super fiber quality.

Secondly, elite germplasms could be selected as parents in the breeding program based on the association results; the typical carrier materials aggregated the allelic variation with the most positive efficiency, more distant genetic relationship and complementary elite and stable genetic traits. For example, according to the phenotypic effects of each germplasm based on the BLUP of six fiber quality traits in eight environments (Additional file 6), ZY495, ZY488 and ZY64 had higher phenotypic effects for FUHML, FS and MV, respectively; meanwhile, ZY43 had higher phenotypic effects for FUHML and relatively lower phenotypic effects for FS. Comparing the genetic similarity coefficients of ZY495, ZY488 and ZY64 to ZY43, the lowest genetic similarity coefficient between ZY64 and ZY43 indicated that they could be selected to improve fiber quality.

## Conclusions

An association mapping population of 503 nation-wide upland cottons in China was genotyped by 494 genome-wide SSRs and phenotyped in eight environments, which revealed abundant molecular diversity and phenotypic variations. The population was divided into seven subpopulations by comprehensive analysis; and the attenuation distance of LD in this population was 0–5 cM. MLM based association mapping for fiber quality detected known and novel elite alleles, and typical materials were identified. The results in this study will provide a platform for future genetics and breeding in cotton.

## Methods

### Plant materials

A set of 503 upland cotton inbred cultivars (Additional file 3) constructed the mapping population panel, which represents diverse genetic resources related to fiber quality traits. These inbred cultivars were mainly collected from China and two foreign countries and were divided into seven cotton growing regions according to different ecological characteristics, including 79 from the NNIR, 28 from the NSEMR, 225 from the YRR, 141 from the YtRR, 4 from the SC, 20 from the United States, and 6 the Soviet Union. These inbred cultivars are frontier specimens for cultivar development in China. The cultivars in the population panel covered four variety improvement stages: the preliminary phase of the *G. arboreum* cotton and upland cotton improvement (1920s–1950s);, the breeding stage of increasing yield (late 1950s–1970s); the breeding stage of comprehensive improvement of yield, fiber quality and disease resistance (1980s–1990s); and the breeding stage of good fiber, genetically modified and mechanized harvesting cotton varieties (1990s–2010s).

The 503 upland cotton cultivars were collected from the Institute of Cotton Research of Chinese Academy of Agricultural Sciences (Anyang, China), Xinjiang Academy of Agriculture and Reclamation Science (Shihezi, China), Shihezi Academy of Agricultural Sciences (Shihezi, China), Henan Academy of Agricultural Sciences (Zhengzhou, China), Jiangsu Academy of Agricultural Sciences (Nanjing, China), Cotton Research Institute of Shanxi Aacademy of Agricultural Sciences (Yuncheng, China), Shandong Cotton Research Center (Jinan, China), and Hebei Agricultural University (Baoding, China). All the cultivars were authorized for only scientific research purpose, and were deposited in the original institutes and Huazhong Agricultural University.

### Field experiments and phenotype data collection

Field experiments were performed in Shihezi (SHZ), North Xinjiang (E85.94°, N44.27°, the NIR), Kuerle (KEL), South Xinjiang (E86.06°, N41.68°, the NIR), Yuanyang (YY), Henan (E113.97°, N35.05°, the YRR), and Huanggang (HG), Hubei (E114.87°, N30.44°, the YtRR) in 2012 and 2013. These locations are included in the main cultivated cotton areas covering the cotton ecological areas. The upland cotton cultivars we collected are not endangered or protected species. The field works and sampling were authorized by local governments, respectively.

Standard field plots and agronomic technologies were used to grow the populations in eight environments (in two years and four locations); and field trials followed a completely randomized block design with two replicates in each environment. Taking into consideration of the different cultivation management patterns in four locations, the designs of the field experiments were conducted as follows: using a specific wide-narrow distance planting pattern, the row spacing was (40 + 50 + 46) cm, with 9.5 cm between individuals and 50 individuals per 5 m in one row, planted by sowing in a hole and dripping irrigation in Xinjiang (both in Shihezi and Kuerle); the row spacing was 90 cm, with 30 cm between individuals and 12 individuals per 5 m in one row, planted by sowing in hole and flooding in Yuanyang; the row spacing was 100 cm, with 40 cm between individuals and 10 individuals per 5 m in one row, seedling transplantation and without irrigating in Huanggang.

To measure the fiber quality traits, 20 bolls were collected from the middle fruit branches of plants in each line. After grinning, 10–15 g fibers of each sample were sent to the Institute of Cotton Research, Shihezi Academy of Agricultural Sciences to test the fiber quality. The fiber quality-related traits were tested at 20 °C, and 65 % relative humidity with an HVI1000 Automatic Fiber Determination System, including the fiber upper half mean length (FUHML), fiber strength (FS), micronaire value (MV), fiber uniformity (FU), short fiber (SF), and fiber elongation (FE).

### Phenotype data analysis

The fiber quality traits statistics were obtained from one-year-one-location and eight environments by best linear unbiased prediction (BLUP) [50]. Broad-sense heritability was calculated for each trait according to the method described by Liu et al. [51], which refers to the percentage of the total variance of phenotype of genetic variation, or phenotype variance of genetic variance percentage.

Analysis of phenotypic changing trends and relevance of fiber quality traits were shown in boxplot form, and the correlation pictures between the environment and environment by the different code were drawn using the “R” program.

SPSS 17.0 software (http://www-01.ibm.com/software/analytics/spss/) was used to calculate basic statistics, including extreme values, mean value, standard deviation, variable coefficient, and the statistics for the correlation between traits.

### SSR markers genotyping and genetic diversity

A total of 494 SSR markers were selected at an average of 10 cM from the interspecific genetic map constructed with 5152 markers in cotton [12] and were used to screen the genetic polymorphism of the population. Total DNA was extracted from young leaves using the modified CTAB method [52]. Polymerase chain reaction (PCR), electrophoresis (6 % denaturing polyacrylamide gel electrophoresis for SSR and 8 % non-denaturing polyacrylamide gel electrophoresis for SSCP) and silver staining were performed according to the methods described by Li et al. [52]. SSR and SSCP fragments were coded as “1” for present, “0” for absent, and “?” for missing data.

The number of alleles loci, number of genotypes, gene diversity, and polymorphism information content (PIC) of polymorphic markers were calculated by Powermarker software 3.25 [53] (http://tree.bio.ed.ac.uk/software/figtree/). Genetic differentiation (PhiPT) was estimated using the AMOVA and the Frequency function of GENALEX 6.2 software [54] (http://biology.anu.edu.au/GenAlEx/). The hierarchical diagram and matrices of genetic similarity were obtained from the NTSYS-pc version 2.10e statistical package based on Jaccard’s algorithms [55].

### Population structure

Unrooted tree and circular clustering figure were generated on the basis of the gene frequency, genetic distance, and analysis of neighbor-joining (NJ) trees from Powermarker software 3.25 [53]. A two-dimensional diagram of principal coordinates analysis (PCA) was produced based on a genetic distance (GD) matrix by GENALEX6.2 software [54].

The population structure was analyzed and evaluated for further association mapping by STRUCTURE version 2.1 [31, 32] using an admixture model; MCMC was set to 50000, burn-in was set to a running time of 100000 and the K value was set to 2–10. Each K value was repeated five times. To select a suitable K, drawing LnP(D) and K values-changing trend diagrams were used to determine which K value reached the largest plateau of the Ln P(D) values. If there was no such plateau, then the drawing delta (K) and K values-changing trend diagrams were used to judge which inflection point of the K value was the most suitable for the number of the population [56]. Cluster analysis, principal component analysis (PCA) and structure analysis were used to determine a reasonable population structure and correct false positives caused by the population structure for association mapping in this study.

### Linkage disequilibrium and LD decay

The distribution diagrams of the genome-wide linkage disequilibrium (LD) attenuation were drawn in SPSS statistics17.0 software (http://www-01.ibm.com/software/analytics/spss/) based on r^2^ and D’ from the results of the LD running in TASSEL [57] and the genetic distance. The LD decay scatter plot shows the relationship between r^2^ and D’ on the y-axis and the genetic distance between marker pairs on the x-axis to understand the pattern of LD in the various annotation groups or entire genome [58].

### Association analysis

The existence of the population structure and relative kinship in the population always result in a high level of spurious positives in association mapping. In this study, the TASSEL V2.1 [57] software package was used to analyze the molecular marker data and the phenotypic data of the fiber quality in eight different environments individually using the three models of GLM (P + G + Q and P + G + PCA) and MLM (P + G + Q + K). The Q matrix was derived from Clumpp software [59] to merge five repetitive results up to the K value for seven using the Structure software package. Kingship was generated from the TASSEL software package results. The P values of markers associated with QTL were regulated by the method of multiple testing correction by controlling the false discovery rate [60].

### Elite allele exploration and gene-related functional annotation

According to the results of the genome-wide analysis study, the primer sequences of the QTL that were mapped on a single chromosome, repeated in multiple environments and reported the same as previous studies were considered to compare with the *G. arboreum* [41], *G. raimondii* [42] and *G. hirsutum* [43] genome sequences, and then internal gene spacer and gene annotation including the function and pathway were discovered.

### Ethics (and consent to participate)

Not applicable.

### Consent to publish

Not applicable.

### Availability of data and materials

All relevant data are available within the manuscript and its additional files.

## Additional files

Additional file 1: Table S1. The SSR markers information of 503 cultivars. (XLSX 22 kb) Additional file 2: Figure S1. Clustering analysis tree of 503 *G. hirsutum* cultivars based on Jaccard’s similarity coefficients by SSRs. (PDF 27 kb) Additional file 3: Table S2. The pedigrees origin information of 503 cultivars. (XLS 124 kb) Additional file 4: Figure S2. Neighbor-joining trees of 503 *G. hirsutum* cultivars. Dendrogram of 503 *G. hirsutum* cultivars by NJ analysis in the form of unrooted tree. (a): Based on Nei’s genetic distance, the population formed 7 distinct groups from I to VII, including 106, 19, 147, 67, 9, 103, and 52 cultivars, respectively; (b): Dendrogram of 503 *G. hirsutum* cultivars by NJ analysis in the form of round tree. Colors in the dendrogram correspond to the population from seven different cotton regions. (TIF 5688 kb) Additional file 5: Figure S3. LD decays as a function of genetic distance in the association panel consisting of 503 upland cotton cultivars. (a): LD decays within distance in the form of r^2^ based on linkage disequilibrium loci on the same chromosome; (b): LD decays with distance in the form of r^2^ based on different intervals of linkage disequilibrium loci on the same chromosome; (c): LD decays with distance in the form of D’ based on linkage disequilibrium loci on the same chromosome. (TIF 2615 kb) Additional file 6: Table S3. The BLUP of six fiber quality traits of 503 cultivars. (XLS 243 kb) Additional file 7: Table S4. Phenotype statistics of six fiber quality traits in the tested cotton cultivars. (XLSX 12 kb) Additional file 8: Table S5. The correlation analysis of six fiber quality traits. (XLSX 10 kb) Additional file 9: Figure S4. Quantile–quantile plots of estimated–log10(P-value) from association analysis using three models. (a) FUHML; (b) FS; (c) MV; (d) FU; (e) SF; (f) FE. The black line is the expected line under the null distribution. Under the assumption that there are few true marker associations, the observed P values are expected to nearly follow the expected P values. (TIF 8527 kb) Additional file 10: Table S6. The marker loci related to six fiber quality traits of phenotypic variation. (XLSX 18 kb) Additional file 11: Table S7. The results of comparison of the allelic variation loci related fiber quality and the *Gossypium arboreum* genome. (XLSX 14 kb) Additional file 12: Table S8. The results of the comparison of the allelic variation loci-related fiber quality and the *Gossypium raimondii* genome. (XLSX 14 kb) Additional file 13: Table S9. The results of the comparison of the allelic variation loci-related fiber quality and the *Gossypium hirsutum* genome. (XLSX 14 kb) Additional file 14: Table S10. The SSR marker loci associated with six fiber quality traits of phenotypic effects and typical materials. (XLSX 15 kb)

## Abbreviations

BLUP: best linear unbiased prediction
FE: fiber elongation
FS: fiber strength
FU: fiber uniformity
FUHML: fiber upper half mean length
LD: linkage disequilibrium
MLM: mixed linear model
MV: micronaire value
NIR: Northwestern Inland Region
NSEMR: Northern Specific Early Maturation Region
QTL: quantitative trait loci
SCR: South China Region
SF: short fiber
SSR: simple sequence repeat
YRR: Yellow River Region
YtRR: Yangtze River Region
