# Supplementary figures and images for: Genome-wide SSR-based association mapping for fiber quality in nation-wide upland cotton inbreed cultivars in China
Source: BMC Genomics. 2016 May 13;17:352. doi: 10.1186/s12864-016-2662-x (PMC4866303; doi:10.1186/s12864-016-2662-x)

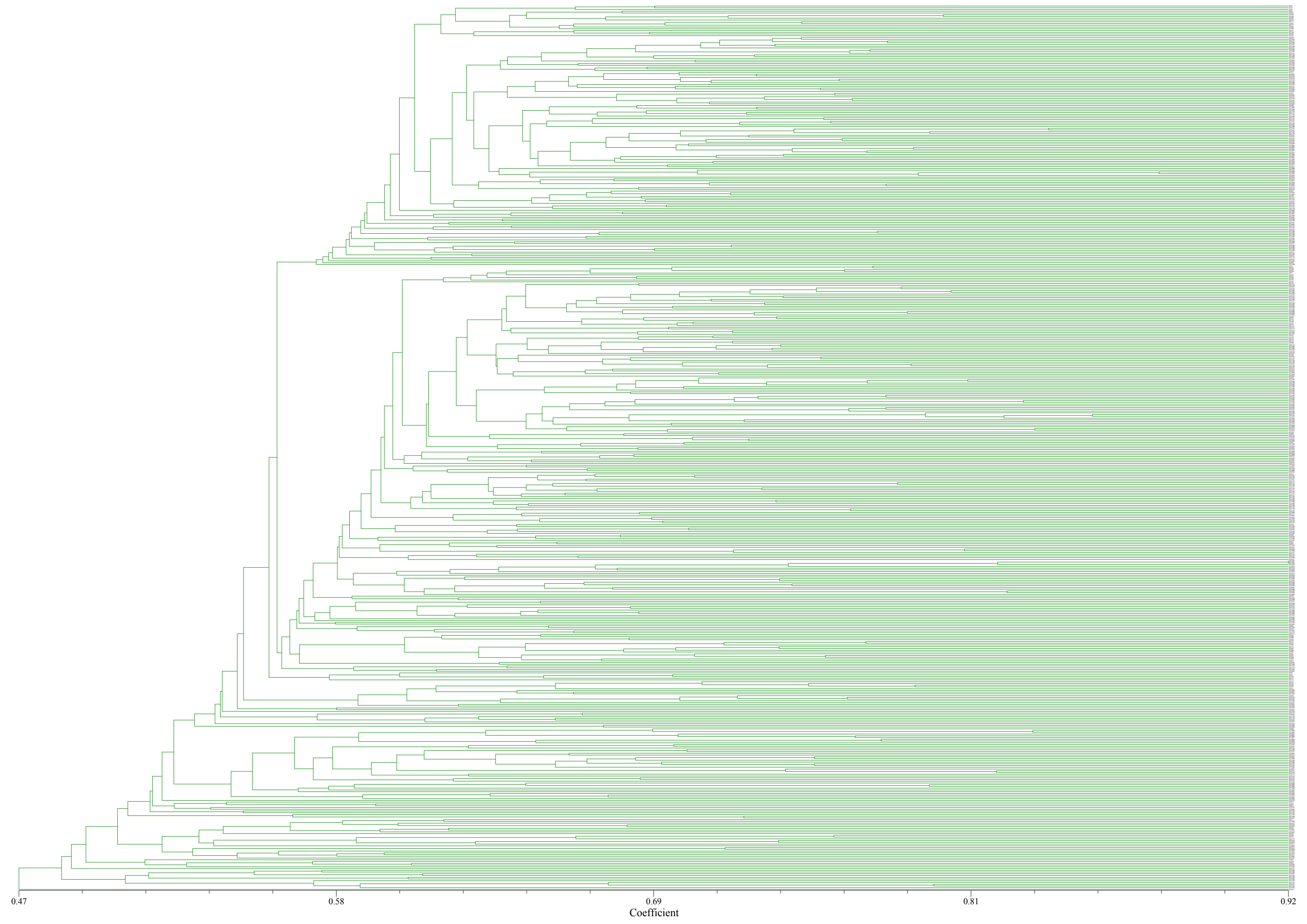

Supplement: Additional file 2: Figure S1. — Clustering analysis tree of 503 G. hirsutum cultivars based on Jaccard’s similarity coefficients by SSRs. (PDF 27 kb) [file 12864_2016_2662_MOESM2_ESM.pdf]
